# [Retracted] Treatment with harmine ameliorates functional impairment and neuronal death following traumatic brain injury 

**DOI:** 10.3892/mmr.2026.13883

**Published:** 2026-04-20

**Authors:** Zeqi Zhong, Yuan Tao, Hui Yang

Mol Med Rep 12: 7985–7991, 2015; DOI: 10.3892/mmr.2015.4437

Following the publication of the above paper, it was drawn to the Editor's attention by a concerned reader that, in addition to the apparent duplication of western blot data both within Fig. 6A and comparing Fig. 5A with Fig. 8A in the paper itself, western blot data featured in Fig. 5A were strikingly similar to data that had already been submitted for publication to the journal *Molecular Medicine Reports* in different form in an article written by different authors at different research institutes. In addition, western blot data featured in Figs. 6A and 8A also reappeared in a paper written by different authors at different research institutes that was submitted for publication at a later date to the journal *Experimental and Therapeutic Medicine.*

In view of the fact that the abovementioned data had already apparently been submitted for publication before this article was received at *Molecular Medicine Reports*, the Editor has decided that this paper should be retracted from the Journal. The authors were asked for an explanation to account for these concerns, but the Editorial Office did not receive a reply. The Editor apologizes to the readership for any inconvenience caused.

